# From harmful nutrients to ultra-processed foods: exploring shifts in ‘foods to limit’ terminology used in national food-based dietary guidelines

**DOI:** 10.1017/S1368980022002580

**Published:** 2023-11

**Authors:** Kim Anastasiou, Patricia Ribeiro De Melo, Scott Slater, Gilly A Hendrie, Michalis Hadjikakou, Phillip K Baker, Mark Andrew Lawrence

**Affiliations:** 1 School of Exercise and Nutrition Sciences, Deakin University, Building J, Holland Ave, Burwood, VIC 3125, Australia; 2 Health and Biosecurity, CSIRO, Australia; 3 Centre for Integrative Ecology, School of Life and Environmental Sciences, Deakin University, Melbourne, VIC, Australia; 4 Institute of Physical Activity and Nutrition, Deakin University, Geelong, Australia

**Keywords:** Dietary guidelines, Nutrition policy, Nutrition terminology, Discretionary foods, Ultra-processed foods, Foods to limit

## Abstract

**Objective:**

The choice of terms used to describe ‘foods to limit’ (FTL) in food-based dietary guidelines (FBDG) can impact public understanding, policy translation and research applicability. The choice of terms in FBDG has been influenced by available science, values, beliefs and historical events. This study aimed to analyse the terms used and definitions given to FTL in FBDG around the world, including changes over time and regional differences.

**Design:**

A review of terms used to describe FTL and their definitions in all current and past FBDG for adults was conducted, using a search strategy informed by the FAO FBDG website. Data from 148 guidelines (96 countries) were extracted into a pre-defined table and terms were organised by the categories ‘nutrient-based’, ‘food examples’ or ‘processing-related’.

**Setting:**

National FBDG from all world regions.

**Participants:**

None.

**Results:**

Nutrient-based terms (e.g. high-fat foods) were the most frequently used type of term in both current and past dietary guidelines (91 %, 85 %, respectively). However, food examples (e.g. cakes) and processing-related terms (e.g. ultra-processed foods) have increased in use over the past 20 years and are now often used in conjunction with nutrient-based terms. Regional differences were only observed for processing-related terms.

**Conclusion:**

Diverse, and often poorly defined, terms are used to describe FTL in FBDG. Policymakers should ensure that FTL terms have clear definitions and can be integrated with other disciplines and understood by consumers. This may facilitate the inclusion of the most contemporary and potentially impactful terminology in nutrition research and policies.

Food-based dietary guidelines (FBDG) have played a key role in food policy and nutrition education since the mid-20th century. FBDG provide advice to professionals and the population on the recommended food group intake and dietary patterns and are used to inform the development of food and nutrition policies and programmes, such as school nutrition curriculums and institutional food provision policies^(1)^.

Nutrition science plays an instrumental role in the development of FBDG, as nutrition guidance should be informed by the latest scientific findings^(2,3)^ and align with local and international nutrition targets and guidelines. More recently, these have moved beyond malnutrition and chronic disease prevention to encompass issues such as environmental, social and economic sustainability^(4,5)^. However, the framing of public health nutrition problems by policymakers does not occur in a social and political vacuum^(6)^. In fact, what constitutes relevant evidence to inform the making of FBDG is not determined by science alone, but unavoidably influenced by prevailing views of the time^(2)^ and the values and beliefs of those involved in FBDG development^(3)^. Historically, nutrition science agendas have been influenced by prevailing interests, views^(2)^ and available funding^(7)^. Thus, FBDG can offer insights into both the evolution of the scientific understanding of nutrition and how the framing of nutrition problems has changed over time.

Effective terms and definitions used in FBDG may provide clarity for the target audience (consumers, policymakers and researchers) on what is viewed as ‘healthy’ or ‘unhealthy’. Nutrition science is already plagued by public confusion resulting from non-expert promotion of alternative ‘fad’ diets, sensational news reporting of individual studies, persuasive advertising of unhealthy products by food companies and, necessarily, by ongoing scientific discovery leading to changes in the underlying evidence base for dietary advice^(8)^. Frequently changing terminology may lead to further difficulty in policymaking, and subsequent confusion among the public, thus clarity should be a priority for FBDG committees.

National FBDG recommendations can be made in the context of two types of nutrition exposures: nutrients or foods^(9)^. Broadly, recommendations informed by nutrient exposures tend to be reductionist in scope, as they focus on isolated nutritional components of foods^(10)^. These types of terms are often supported by forms of evidence such as randomised controlled trials^(8)^. On the other hand, recommendations based on food exposures are often informed by a holistic view of food^(10,11)^, which includes looking at the broader contextual aspects of food products, such as the role of food in diets, their level of processing and purpose in social contexts^(11)^. Frequently, these food-based terminologies are underpinned by evidence derived from observational studies^(12)^, which are in turn ranked as lower quality evidence when compared to those derived from randomised controlled trials^(13)^. As a result, the ‘evidence hierarchy’, whereby studies are ranked and graded according to their methodology type, may also influence the terms used in FBDG.

Choices on which term is used to describe ‘foods to limit’ (FTL) can be particularly contentious because recommendations to consume less of a particular food may influence intake and in turn the popularity and potentially the sales of certain products^(3)^. It has been suggested that companies manufacturing foods ‘targeted’ by dietary guidelines tend to prefer nutrient-based terminology because it shifts the focus from their food product to nutrients that are present in many different foods^(14)^, meaning any advice to limit specific products is less direct. The focus on nutrient composition rather than whole food choices can provide opportunities for manufacturers to position their existing products within FBDG recommendations by manipulating those products’ nutrient profiles rather than risk losing market share, or from subsequent policy actions such as marketing restrictions that may reduce absolute levels of consumption. Previous studies have found that nutrient-based terms result in poorer, decontextualised comprehension of FBDG messages by the general public, compared with more tangible examples of real foods^(15)^.

Previous reviews have analysed the types of nutrients targeted by FBDG^(16)^ or the uptake and communication of processing terms^(17)^. A recent review has also analysed the suitability of FTL terminology for policy application, finding that no existing term or definition is suitable for all purposes^(18)^. However, some terms, such as ‘unhealthy food and drinks’, were better aligned with a wide variety of policy applications, measured against the nourishing framework^(18)^.

Research analysing the variety of terms used to describe FTL and their progression over time is limited. This is relevant because studies that analyse changes over time may reveal trends in terminology and provide insights into the prevailing views and historical factors that have helped shape food and nutrition guidance to date. Providing insight on these variables is important as this can help future FBDG understand how to position their language to ensure clarity for consumers, policymakers and researchers. Therefore, the present study summarises the terms and definitions used to describe FTL in national FBDG, including temporal and regional trends over time. This research is intended to provide greater clarity and guidance about the types of terms that can be adopted in FBDG.

## Methods

The search for guidelines occurred between January and November 2021. The FAO FBDG website provides a summary of past and present official national FBDG^(1)^ and was used to inform the countries used in the search strategy, as outlined below.

Both past and current FBDG were searched initially on each country’s page on the FAO FBDG website. If FBDG were not located, then Google was searched (the first 100 results were reviewed), followed by the Deakin University library service and WorldCat^(19)^. If these processes were unsuccessful in locating the relevant document, requests were sent to the publishing institution(s). Google search strings followed the following format: country name, year of guideline publication, and either the words ‘food-based dietary guidelines’, or the name of the guidelines (if stated on the FAO website). Original records of past FBDG were more challenging to find, therefore official websites, peer-reviewed publications or documentation provided by the publishing organisation which stated the key FBDG messages were also included.

Countries often provide two sets of FBDG documents; a background document that provides information on the development of the FBDG and/or scientific rationale for guidelines and a consumer document that provides summaries of the guidelines in a way that is either designed for professionals promoting dietary guideline messages or for the public. As the purpose of this study was to analyse FBDG messages (not evidence), the most comprehensive consumer-oriented document was included. FBDG which were published in a language other than English were translated using Google Translate. FBDG which focussed on particular population groups other than adults (e.g. children) were excluded. FBDG specific to health conditions (e.g. type 2 diabetes) were also excluded.

Results from all past and current FBDG were recorded in a pre-defined table (see online Supplemental Tables S1 & S2). Data were extracted to inform the analysis of: (1) the terms used in current FBDG, (2) changes in terms used over time and (3) regional differences in terms used.

Headline messages related to FTL were defined as any food, beverage or nutrient which the FBDG recommended to reduce, exclude or moderate consumption in the FBDG introduction, conclusion, title or figures (such as a food pyramid or plate). In FBDG which did not have ‘headline messages’, all messages relating to FTL were included. Definitions of FTL were also recorded, where a statement of the exact meaning of the term was provided. Descriptions of biochemical functions, health implications or lists of foods were not considered definitions as they did not provide clarity on the rationale for including or excluding specific foods in the FTL message(s). Other data extracted included the guideline’s year of publication, international organisations offering technical or financial support in guideline development and the world region of the home country.

FBDG were subsequently divided according to the type of term used to describe the FTL. These groupings were adapted from Hadjikakou and Baker^(20)^ and classified based on the use of three distinct types of terminologies: nutrient-based terms (e.g. *foods high in fat, salt and sugar; salt, sugar, fat*), food examples (e.g. *soft drinks, confectionery*), and processing-related terms (e.g. *highly processed foods, ultra-processed foods)*.

These data were explored in the discussion in the context of potential influences on the choice of FTL terms, how well consumers understand the term, the use of clear supporting definitions and their ability to deliver on multi-sectoral food system goals.

## Results

The FAO website contains the details of the FBDG of ninety-five countries and a total of ninety-six current FBDG, because Belgium has two official FBDG. However, not all were accessible or translatable. The FAO website, in combination with the additional searching, resulted in eighty-six current and sixty-two past FBDG available for inclusion in this review.

### Terms used in current food-based dietary guidelines

All current FBDG explicitly mentioned FTL but only 22 % (19/86) defined the FTL term. A summary of the terms used in current FBDG is presented in Table 1, where the examples of nutrient-based and processing-related terms are listed in order of most to least frequently used. Full details of the terms used in all FBDG are available in Supplemental Tables S1 and S2.

**Table 1:** Terms used to describe ‘foods to limit’ in current national FBDG

Type of term	*n*	%	Examples of terms	Definition provided *n*	%	Definition example(s)	Date range
Nutrient-based terms	78	91	• Sugar/sugary foods and drinks/foods and drinks high in sugar • Salt/Na/foods high in salt • Fat/fatty foods/foods high in fat • Alcohol • Saturated fats • Trans fats • Cholesterol • Discretionary choices • Empty calories	13	17	*“Empty calories… are not necessary for a balanced diet and can even harm your health if taken too much”* ^(49)^. *“Discretionary choices are high in saturated fat (natural or added) and/or added sugars or salt or alcohol. These foods and drinks can contribute many kilojoules and displace other more nutritious foods from the diet. Many have low levels of essential nutrients”* ^(48)^.	1991–2021
Food examples	60	70	• Sugary drinks • Butter and margarine • Condiments • Meat products • Red meat • Fried foods • Snacks • Instant soups • Alcoholic beverages • Beverages	2	3	*“Alcoholic beverages contain ethyl alcohol or ethanol, supply energy – 7 kcal or 29 kJ/g ethanol, but do not provide essential nutrients…”* ^(50)^	1991–2021
Processing-related terms	22	26	• Ultra-processed foods • Highly processed foods • Processed meats • Processed foods • Processed food and drinks • Processed sauces • Processed foods rich in salt, sugar and fats	5	22	*“Ultra-processed foods are industrial formulations made entirely or mostly from substances extracted from foods… derived from food constituents… or synthesised in laboratories from food substrates or other organic sources…”* ^(34)^	2008–2020
Total	86 FBDG						

FBDG, food-based dietary guidelines.

**Fig. 1:**
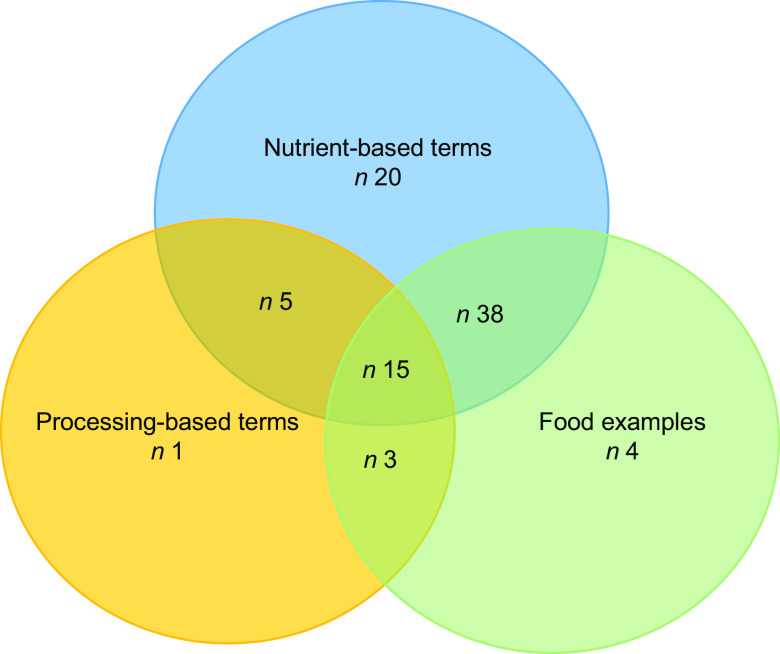
Venn diagram displaying the use of one type or multiple types of terms within current FBDG (total FBDG *n* 86). FBDG, food-based dietary guidelines

Nutrient-based terms were most frequently used to describe FTL in current FBDG (*n* 78/86, 91 %). Of note is that the majority of FBDG documents utilised more than one type of term (*n* 61/86, 71 %). Some FBDG even utilised a mix of terms within the same guideline. For example, the 2011 Indian Dietary Guidelines recommended people limit ‘edible oils, animal foods, ghee, butter, vanaspati, salt, processed foods rich in salt, sugar and fats’, utilising food examples, nutrient and processing-related terminology. The most common combination was the use of nutrient-based terms with food examples (*n* 38/86, 44 %). The overlap between terms used in current FBDG is displayed in Fig. 1.

### Changes over time

**Fig. 2:**
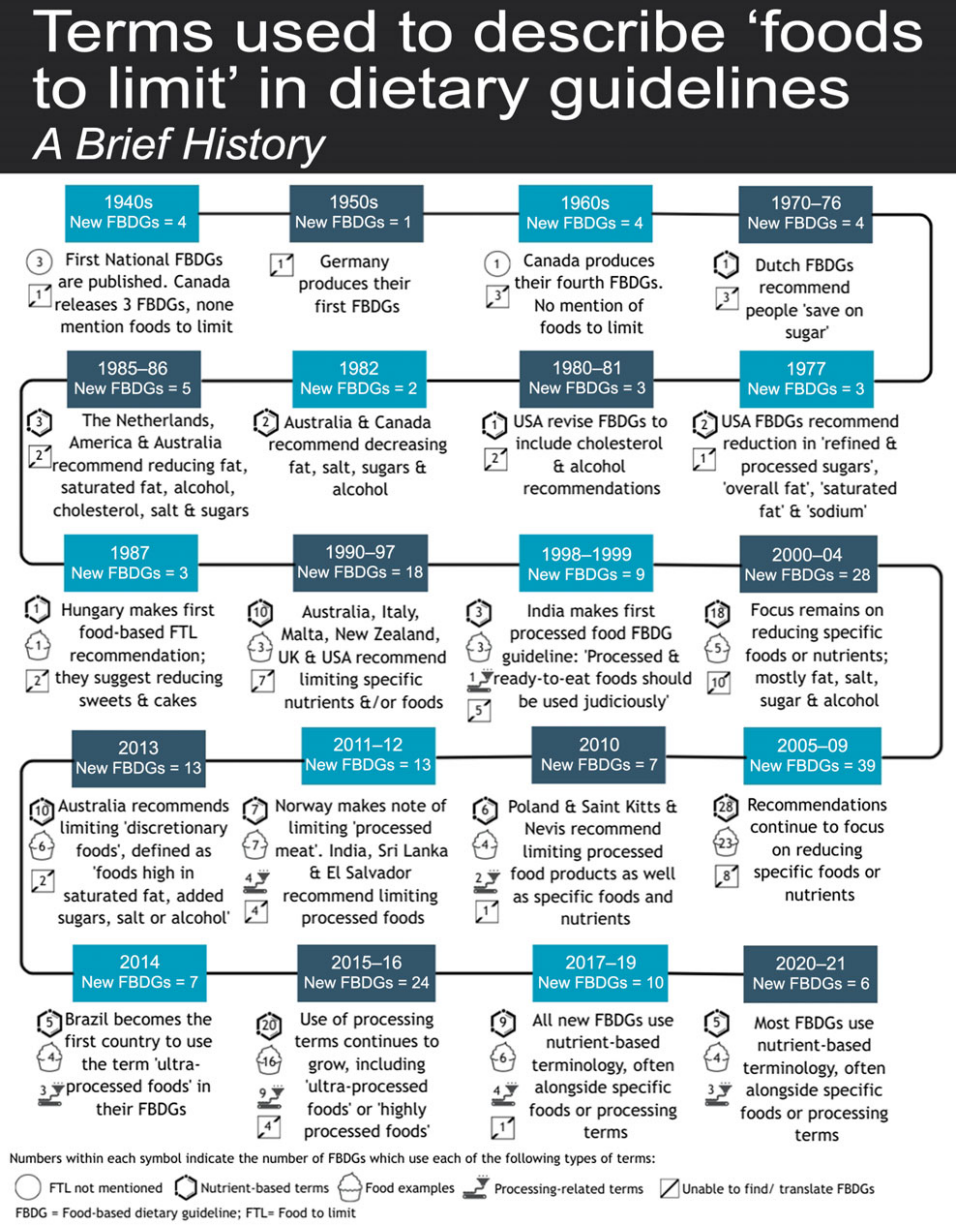
A brief history of terms used to describe ‘foods to limit’ in national food-based dietary guidelines. Created using VISME.com

**Fig. 3:**
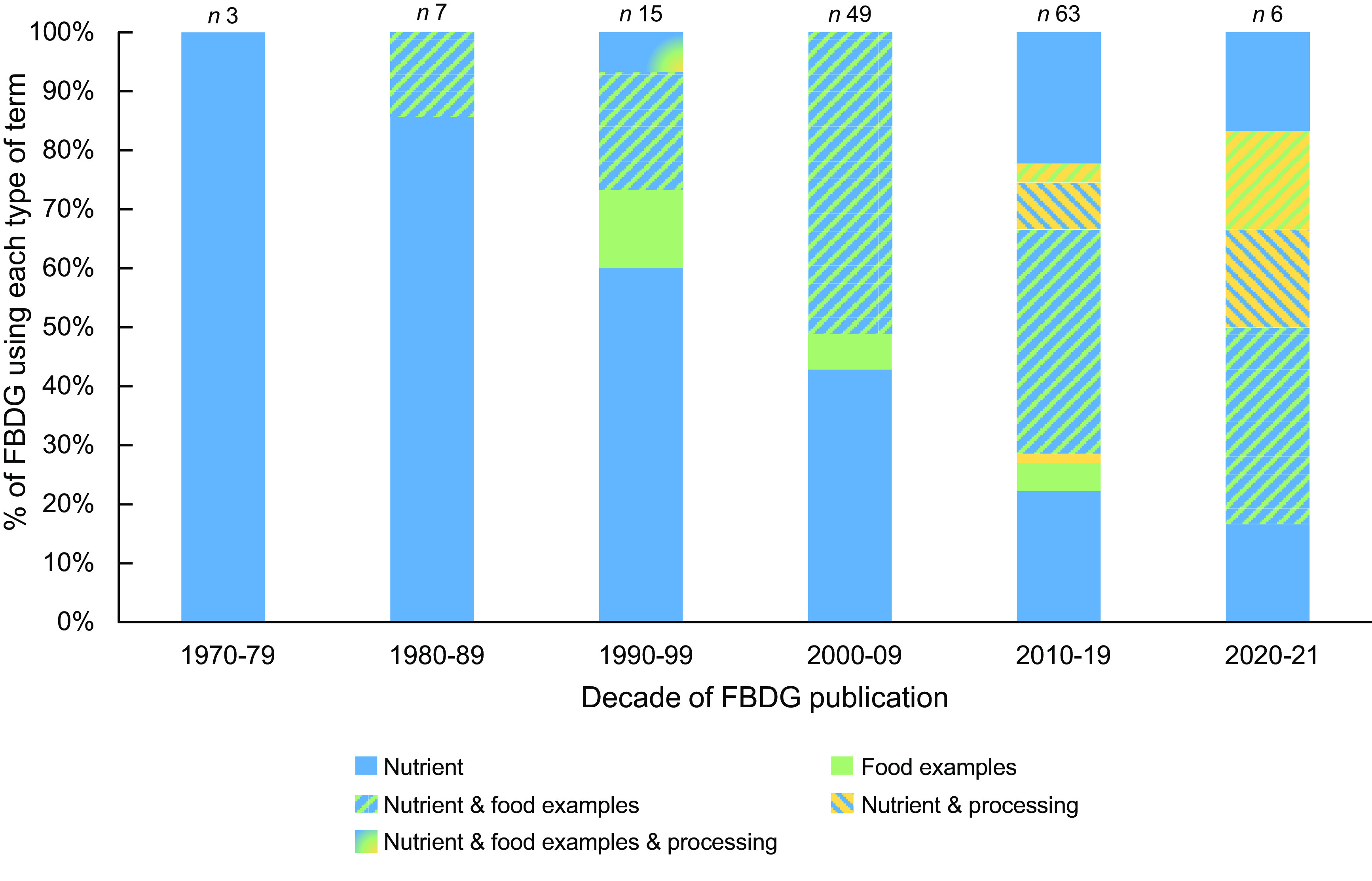
Changes in types of terms used to describe ‘foods to limit’ in FBDG since the introduction of ‘foods to limit’ in the 1970s. FBDG, food-based dietary guidelines

Figures 2 and 3 show changes in terms used to describe FTL in FBDG over time based on the 148 FBDG documents included. Detailed information on the FTL terms and definitions used in each FBDG can be found in Supplemental Tables S1 and S2.

The first country to include a message about limiting foods or nutrients in their FBDG was Denmark, which in 1976 recommended Danish people ‘save on sugar’. Nutrient-based recommendations continued to dominate FTL messages in the late 1970s and 1980s. In 1987, Hungary made the first recommendation to limit specific foods; *‘We only eat sweets and cakes once a week as a finishing touch to meals, never between meals’*. This was the first headline FTL guideline identified in this analysis that did not use a nutrient-based term. The terms used in the 1990s and 2000s were mostly nutrient-related (usually fat, saturated fat, salt, sugar, alcohol) or food-related (such as fried foods, butter, margarine, snacks, sodas, candy bars, oil, meat and eggs) (see Fig. 2).

In 1998, the *Dietary Guidelines for Indians* produced the first headline FBDG message related to processed foods, which stated ‘*Processed and ready-to-eat foods should be used judiciously.*’ Processing language was not used again in FBDG until 2011 when Norway recommended its citizens ‘*limit the intake of red meat and processed meat’*.

Processing-related terminology has increased since the release of the 2014 *Dietary Guidelines for the Brazilian Population* (see Fig. 2), which was the first FBDG to use the term ‘*ultra-processed foods’* and its associated definition. The terms *‘ultra-processed foods’* or ‘*highly-processed foods’* have been used in eight out of thirty-nine FBDG published since 2014. Other processing-related terms, such as *‘processed foods’* and ‘*processed meats*’, have been used in an additional eight guidelines (out of thirty-nine). Nutrient-based terms were the most common type of term in past FBDG (*n* 52/62, 80 %) and continue to be the most common terms used in the FTL section of FBDG messages (used in 56/63, 88 % of the FBDG published since 2011), but are now often used in conjunction with specific food examples or processing terms (see Figs. 2 and 3).

### Regional differences in terms used to describe ‘foods to limit’ in food-based dietary guidelines

**Fig. 4:**
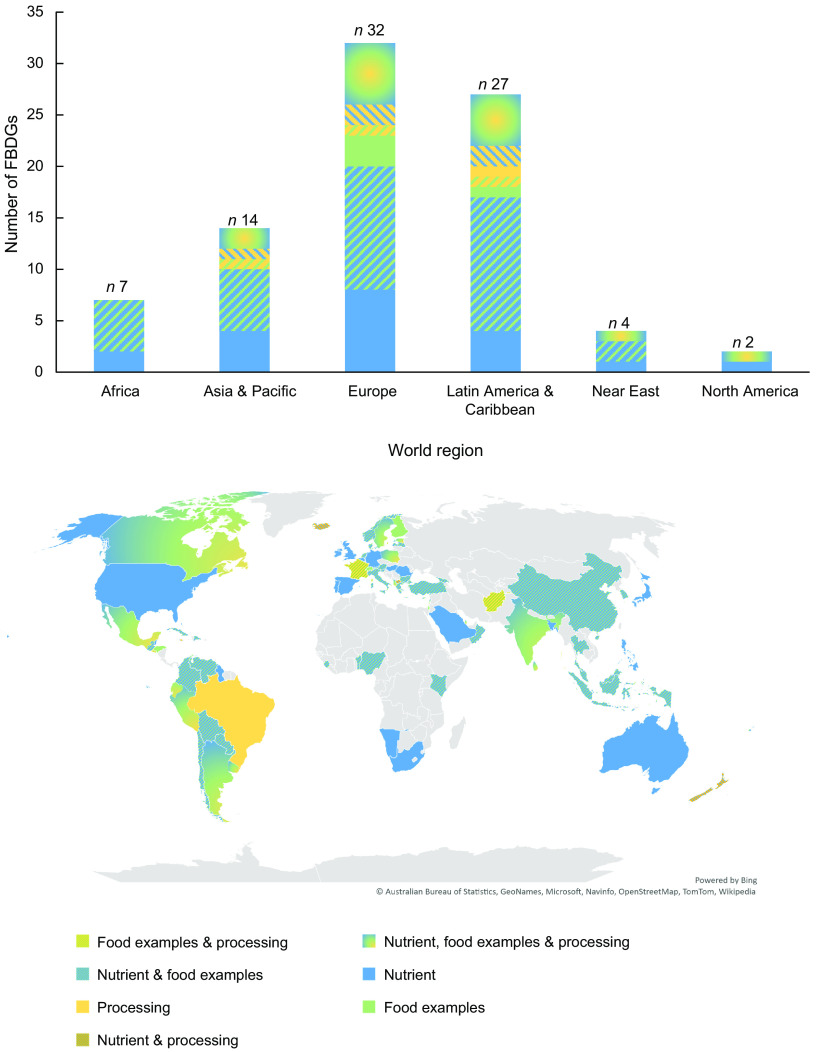
Regional differences in terms used to describe ‘foods to limit’ in current FBDG. FBDG, food-based dietary guidelines

All world regions utilised nutrient-based terms and food examples in their FBDG. The use of processing-related terms was more region-specific (Fig. 4). Processing-related terms were used in approximately one-third of the current FBDG in Europe (9/32), Asia (4/14) and Latin America and the Caribbean (9/27). Only one out of four FBDG in the Near-East region (*n* 1/4) and none of the African FBDG (*n* 0/7) used processing-related terms in their current guidelines.

### Sources of funding and technical support

There was no clear relationship between international agencies which offered funding or technical support and the terms used (see online Supplemental Fig. S1).

## Discussion

This analysis aimed to summarise terms used to describe FTL in FBDG, the changes in terms used over time and regional differences in their use. We found that both past and present FBDG predominantly utilised nutrient-based terms; however, food examples (e.g. soft drinks, confectionery, cakes) and processing-related terms (e.g. ultra-processed foods) have increased in use over the past 20 years. Current FBDG tend to use a mixture of either nutrient-based terms and food examples or nutrient-based terms and processing-related terms.

There are a variety of factors that may have influenced the use of predominantly nutrient-based terminology in FTL FBDG messages. First, and as described in the introduction, is the impact of recent science and ‘evidence hierarchies’ i.e. which evidence is prioritised to inform the FBDG^(6)^. In food and nutrition policy decision-making, the conventional method used to synthesise and translate evidence for policy development has been borrowed from evidence-based medicine, where the quality and strength of evidence is assessed through the use of hierarchy methods^(13)^. As a result, randomised controlled trials, which are well-suited to measuring the effects of isolated nutrients (broadly considered as reductionist terms), are often prioritised over evidence derived from cross-sectional studies on foods or dietary patterns^(8)^. This impacts the messages found in FBDG^(21)^.

Secondly, policy decision-making processes can be influenced by the values, beliefs and interests of policy actors and institutions^(22)^. The choice of members for dietary guideline committees and how questions are framed (e.g. by nutrient, food or dietary pattern exposure) can have substantial impacts on the scope of FBDG and included evidence^(3)^. Thus, the choice of terms and definitions of food groups, as well as the types of nutrition exposure that inform the development of nutritional recommendations around FTL, can be influenced by the world views of those involved in the development of FBDG.

Third, modern nutrition science has been shaped throughout time by a sequence of key historical events^(2)^, such as the Great Depression and the Second World War (where food shortages and nutritional deficiency-related diseases were a major concern amongst the population)^(2,8)^. This led to an ongoing emphasis on nutrients, as found in the results. Together, the above factors influence the terms chosen to describe FTL. The choice of FTL terms has implications for FBDG utility, including the ability of FBDG messages to align with multidisciplinary food system goals, be a communication tool for consumers and provide clarity for researchers and policymakers. The following discussion further explores the historical events which have driven the changes in FTL terms over time and the potential effectiveness of more recent FTL terms.

### Historical events impacting ‘foods to limit’ terminology

FBDG have changed according to population needs and the latest scientific discoveries. Early work in nutrition science focused on discovering nutrients and ensuring the population had enough food to meet their needs and avoid nutritional deficiencies^(2)^. The focus on getting enough food to sustain the population continued through the 1950s and is reflected in the first published FBDG. For example, Canadian FBDG from the 1940s encouraged citizens to make the most of war-time rations, and Germany’s 1956 FBDG were focused on ensuring citizens consumed enough to sustain a labouring workforce for the recovery of a post-war country^(23)^. However, the dramatic shift to industrialised food systems, stemming from the post-World War II paradigm of boosting production and feeding the world at any cost, enabled the overconsumption of unhealthy foods. Subsequently, researchers and citizens began to recognise the long-term health impacts of diets of excess^(14,24)^.

The 1960s ushered in a new era for nutrition science as researchers established links between chronic diseases and overconsumption of specific nutrients^(14)^. Several prominent publications contributed to this; among them, the 1959 publishing of ‘Eat Well and Stay Well’^(25)^ and ‘the Seven Countries Study’^(26)^, two of the seminal works of Ancel Keys. These publications suggested that fat composition in the diet and serum cholesterol levels were universal risk factors for CHD and all-cause mortality. These types of publications likely impacted scientific reports, including FBDG, steering the focus to limiting specific nutrients^(14)^.

The first FTL recommendation appeared in 1976 when the Danish FBDG advised the reduction of sugar. This was followed closely by the first US FBDG in 1977, which targeted total fat, saturated fat, cholesterol, sugar and salt^(27)^. Recommendations to limit nutrients became commonplace as more FBDG emerged in the 1980s, likely due to an ongoing focus on nutrients in nutrition science^(14)^. In addition, there are reports of industry pressure to focus on nutrients, instead of foods^(3)^. For example, the 1977 US FBDG were rapidly revised, due to industry pressure, to encourage the public to ‘*choose meats, poultry, and fish which will reduce saturated fat intake’* instead of the original message, ‘*reduce consumption of meat’* ^(3)^. Nutrient messages were thought to be more palatable for the food industry^(3)^, with previous reports suggesting they enabled companies to reformulate their products and did not overtly encourage consumers to avoid specific foods^(14)^.

From the 1990s onwards, several key global events impacted the rise of FBDG around the world. For example, the International Conference on Nutrition (ICN1), held by the FAO in 1992, was the first global conference specifically focused on nutrition^(28)^. The conference resulted in the ‘World Declaration and Plan of Action for Nutrition’, which recommended that governments disseminate dietary guidance to their country’s population^(28)^. The following decade (2000–2009) saw the largest increase in the number of FBDG published. Countries have cited the ICN1 as the key moment in spurring the development of their FBDG^(29)^. Throughout the late 1990s and early 2000s, FAO and WHO also hosted several workshops and provided support for developing regions to create FBDG^(30)^. As more countries produced FBDG, the use of terms broadened and slowly reflected the ongoing changes in nutrition science.

Statements about restricting specific foods, rather than just nutrients, became more frequent throughout the 1990s and 2000s as more FBDG were produced. FTL listed in FBDG in this period were predominantly high in saturated fat, cholesterol, Na and sugar, such as confectionery, soft drinks, cakes, butter, cured meats, eggs, caffeinated drinks and seasonings. However, recommendations were often softened with the use of words such as ‘moderate’, which may have been poorly understood by the public^(31)^. The increased variety of terms used and foods to target are likely a consequence of the substantial increase in the number of FBDG and a shifting global food landscape that includes a wider variety of readily available processed, packaged foods.

With the latest advances in nutrition science and research methodology, researchers have recognised that nutrients in isolation are less influential on chronic disease outcomes than the combination of foods in diets (including their nutrients, interactions between nutrients and other less measurable components)^(2)^. Nutrient-informed research explains one key mechanism by which foods impact health and, thus, can provide a solid foundation for making recommendations on a broad range of FTL. However, a problem occurs when the nutrient model is used as a stand-alone rationale (without considering the broader contexts of foods and diets) to inform policy interventions and nutrition guidance^(32)^.

Focusing on nutrients in isolation may limit our ability to formulate and implement holistic policy solutions and dietary guidance that could more effectively tackle contemporary public health nutrition problems as well as broader food system issues such as environmental sustainability^(33)^. Instead, researchers and policymakers alike should consider terminology that aligns with the broader concepts of healthy and sustainable food systems.

An example of a more recent term that may address some limitations of nutrient-based terminology is the term ‘ultra-processed foods’. This term first appeared in dietary guidelines in the 2014 Brazilian FBDG, which categorised foods according to the level of processing in a system entitled ‘NOVA’^(34)^. Our results indicate that ‘processing-related’ terms have now been adopted by one-quarter of all current FBDG. Recommendations to limit ‘ultra-processed foods’ have also appeared in guiding documents and global debates such as the 2019 FAO and WHO’s ‘Sustainable and Healthy Diets Guiding Principles’^(35)^ and the 2021 UN Food Systems Summit^(36)^, however, ‘ultra-processed foods’ were not mentioned in the final outcome document.

Ultra-processed foods tend to be high in the nutrients targeted by nutrient-focussed research, namely saturated fat, Na and sugar, but are not defined by the presence or absence of these nutrients. The rationale for limiting these foods in the Brazilian FBDG is as follows.

Because of their ingredients, ultra-processed foods—such as packaged snacks, soft drinks and instant noodles—are nutritionally unbalanced. As a result of their formulation and presentation, they tend to be consumed in excess and displace natural or minimally processed foods. Their means of production, distribution, marketing, and consumption damage culture, social life and the environment^(34)^.

Unlike nutrient-based terms, processing-related terms shift the responsibility away from a heavy focus on consumer choice and towards food production and food environments^(20)^. The term ‘ultra-processed foods’ has been identified as a strong candidate for use in policies but has limited support from food industry stakeholders^(18)^. It has been criticised by those with food manufacturing industry connections for the lack of mechanistic clarity^(37)^; however, mechanistic evidence continues to be a research priority^(38)^, and the evidence base is growing. Most notably, a controlled clinical trial published by Hall *et al*. in 2019 reported a relationship between ultra-processed food consumption and overconsumption of energy, carbohydrates and fat^(39)^. Furthermore, recent observational studies demonstrate a relationship between ultra-processed food consumption and poor health outcomes such as cancer, type 2 diabetes, CVD, irritable bowel syndrome, depression, frailty and all-cause mortality, which have been summarised in previous reviews^(40)^.

### Operationalising terms

In order to be fit-for-purpose, FTL terms need to be able to be integrated into other disciplines to meet global food system targets. They should be clearly understood by consumers and have definitions that provide sufficient clarity for policymakers and researchers.

#### Using FBDG terms to deliver on food system goals

The need for a multi-disciplinary view of food systems has been encouraged to address the cross-sectoral impacts of food production and consumption, and their role in addressing global targets, such as the Sustainable Development Goals and the Paris Climate Agreement^(41)^. This is supported by recent research which shows the multiple environmental and health impacts of food systems, such as the Lancet Commission report on the Global Syndemic of Obesity, Undernutrition and Climate Change^(5)^, and the Eat-Lancet Commission on Healthy Diets from Sustainable Food Systems^(4)^. These ideals were also reflected in the 2021 United Nations Food Systems Summit, which encouraged countries to consider the environmental footprint of proposed diets in FBDG^(36)^. Therefore, to meet international guidelines and targets for healthier and sustainable food systems, terms used by policymakers should be considered for their ability to integrate with other sectors and thus meet multiple sustainable food system objectives.

Terms such as ‘discretionary choices’ and ‘ultra-processed foods’ hold promise for integration with other disciplines. For example, *‘discretionary choices’* aligns with the economic term *‘discretionary consumption’,* which describes the purchasing of non-essential items^(20)^. It also aligns with the concept that scarce natural resources, such as water and land, are being used to create food products that are superfluous to basic human needs, with similar implications in terms of environmental pollutants, such as greenhouse gas emissions^(33)^. However, the term requires clarity as it has been frequently misused in both scientific and policy documents^(18)^.

Alternatively, the NOVA classification could provide a foundation for research and recommendations which integrate nutrition, social and ecological impacts of diets because of its potential ease of integration within food production systems. For example, the levels of processing align well with stages of food production, which is a core component of life-cycle analyses used in environmental sciences. Similarly, analysing foods according to their stages of production could allow social scientists and economists to draw links between safe and satisfactory employment opportunities at different food production stages.

#### Consumer understanding of existing ‘foods to limit’ terms

When choosing terms, FBDG committees should also consider consumer understanding of terminology. Nutrient-based terminology is poorly understood by the public compared with food-based terminology^(15)^, likely because consumers interact and choose specific foods, not nutrients. Simple messages with clear target foods, such as ‘*Drink water instead of sugary drinks’* are more easily understood than nutrient-focused messages, such as ‘*Compare sodium in foods such as soups, breads, and frozen meals and choose the foods with lower numbers’* ^(42)^. Consumers have also expressed difficulty in interpreting messages which specify a quantity of a nutrient (e.g. 30 % fat) or those suggesting a ‘low’ or ‘moderate’ consumption of a nutrient^(31)^.

To the authors’ knowledge, no studies have tested consumer understanding of the term *‘discretionary choices’*. However, a similarly-defined term, ‘extras’, has been interpreted by consumers as anything from snacks to consumption of food items above daily requirements, luxury items or even nutritional supplements^(43)^. ‘*Discretionary choices’* may also be difficult to interpret as it requires an understanding of which foods are not captured by the healthy food groups and are, therefore ‘*discretionary’*.

Preliminary research on consumer understanding of the term *‘ultra-processed foods’* is promising^(18)^. Two separate surveys of consumers found that participants were able to partially define ‘*ultra-processed foods’* and correctly list examples of ultra-processed foods, including some who had not heard the term before^(44,45)^. However, some experienced difficulty differentiating between processed and ultra-processed foods^(44,45)^. Additionally, another review found that few FBDG actually specified the rationale for avoiding ultra-processed food^(17)^, which could provide additional clarity for consumers. FBDG using the term ‘*ultra-processed foods’* should therefore be careful to clarify the differences between ultra-processed and processed foods, and rationale for avoiding them, to avoid unnecessary dietary restrictions. Consumer understanding also needs to be tested in other countries and among a variety of demographics, as current evidence is predominantly from Latin America^(44,45)^.

#### Definitions to support policies and research

FBDG should be careful to provide clear and operational definitions to aid downstream activities, such as designing menus for institutions or for classifying foods in dietary analyses. Avoiding conceptual inconsistencies might also help with research and policy application. In this analysis, we found that less than one-quarter of all FTL terms were defined, and that FBDG often utilised multiple types of terms, sometimes combining them with confusing effects. For example, North Macedonia’s FBDG recommend ‘*Limiting consumption of highly processed foods and beverages high in sugar and fat use’* ^(46)^. This combines and muddles two concepts: nutrient-based terminology and processing-related terminology. The combination of these two terms also narrows the group of products captured, resulting in many foods with associated poor health outcomes being missed. For example, ultra-processed food containing non-nutritive sweeteners would be excluded from this guideline, despite potential health harms^(47)^. Clear and operational definitions should accompany FTL terms to ensure that policymakers and researchers understand the specific foods which are captured by the term. This may also help to reduce conceptual inconsistencies, such as those described above.

### Key recommendations

Our analysis found that more recent FBDG use a mixture of either nutrient-based terms and food examples or nutrient-based terms and processing-related terms. This research is intended to provide greater clarity and guidance about the types of terms that can be adopted in FBDG. In the discussion, we have further interpreted the findings using existing literature to provide context to the factors influencing changes in FBDG FTL terms throughout history. Based on our results and interpretation in the context of previous literature, we encourage policymakers to consider the potential for FTL terms to deliver on the key themes in the discussion, namely, to ensure that:

- Terms are chosen free from industry influence, which has historically led to the preference of reductionist terms which are less well understood by consumers;
- Terms can be applied across multiple disciplines to improve health and sustainability delivered from food systems;
- Terms are accompanied by clear and consistent definitions to ensure consistent interpretation by researchers and policymakers, as well as improved understanding for consumers.

The term ‘ultra-processed foods’ is a recently introduced term for FTL and appears to hold promise when considered against the above criteria but requires further studies to examine how it is understood by different population groups.

### Limitations

To our knowledge, this is the first study to comprehensively examine terms used to describe FTL in FBDG across time. It relied on the use of the publicly accessible FAO FBDG website, which provides a description of and links to ninety-six official FBDG from ninety-five countries around the world. However, access to some FBDG, particularly past FBDG, were limited due to issues with translation, outdated websites or lack of public access.

When undergoing data extraction, only the ‘headline messages’ (defined in the methods section) were extracted, and terms used in the subsequent text were not considered. Usually, terms were consistent across the headline messages and the text, but in some cases, the terms differed and thus not all terms were not captured. Examples include the 2003 Australian FBDG which focussed on nutrients in the headline messages but used the term ‘extra foods’ in the body of the FBDG^(48)^ and the Flemish Dietary Guidelines, which referred to ‘empty calories’ in the headline messages but ‘ultra-processed foods’ in the body of the text^(49)^.

Finally, while we acknowledge the importance of considering foods within the context of dietary patterns, this study was limited to analysing FTL due to the unique challenges relating to their terminologies and definitions.

## Conclusion

This study provides a summary of the terms and definitions used to describe FTL in FBDG, including regional differences and changes over time. FBDG have had a strong historical reliance on nutrient-based terminology, although the use of processing-related terms and food examples has risen over the past 20 years. To provide a strong foundation for consumer understanding, policy translation and future research, dietary guidelines should prioritise using simple terms with clear definitions and avoid the use of conceptually muddled terminology. Deciding which term(s) to use to describe FTL should be built on strong scientific evidence and with a strong focus on contemporary terminology that is most likely to be understood by consumers, supported by definitions that provide clarity for policymakers and researchers. To address broader food systems issues, terms and definitions should enable the integration of research methods with other areas of science, including environmental science, social sciences and economics.

## Supplementary material

For supplementary material accompanying this paper visit https://doi.org/10.1017/S1368980022002580. click here to view supplementary material

## References

[1] FAO (2021) Food-Based Dietary Guidelines. http://www.fao.org/nutrition/nutrition-education/food-dietary-guidelines/en/ (accessed June 2021).

[2] Mozaffarian D , Rosenberg I & Uauy R (2018) History of modern nutrition science—implications for current research, dietary guidelines, and food policy. BMJ 361, k2392. 29899124 10.1136/bmj.k2392 PMC5998735

[3] Nestle M (2013) Food Politics: How the Food Industry Influences Nutrition and Health/M. Nestle. Berkeley, Los Angeles, CA: University of California Press.

[4] Willett W , Rockström J , Loken B et al. (2019) Food in the Anthropocene: the EAT-Lancet Commission on healthy diets from sustainable food systems. Lancet 393, 447–492. 30660336 10.1016/S0140-6736(18)31788-4

[5] Swinburn BA , Kraak VI , Allender S et al. (2019) The global syndemic of obesity, undernutrition, and climate change: the Lancet Commission report. Lancet 393, 791–846. 30700377 10.1016/S0140-6736(18)32822-8

[6] Parkhurst JO & Abeysinghe S (2016) What constitutes “good” evidence for public health and social policy-making? From hierarchies to appropriateness. Soc Epistemol 30, 665–679.

[7] Fabbri A , Lai A , Grundy Q et al. (2018) The influence of industry sponsorship on the research Agenda: a scoping review. Am J Public Health 108, e9–e16. 10.2105/AJPH.2018.304677 PMC6187765 30252531

[8] Mozaffarian D & Forouhi NG (2018) Dietary guidelines and health-is nutrition science up to the task? BMJ 360, k822. 29549076 10.1136/bmj.k822

[9] Tapsell LC , Neale EP , Satija A et al. (2016) Foods, nutrients, and dietary patterns: interconnections and implications for dietary guidelines. Adv Nutr 7, 445–454. 27184272 10.3945/an.115.011718 PMC4863273

[10] Fardet A & Rock E (2014) Toward a new philosophy of preventive nutrition: from a reductionist to a holistic paradigm to improve nutritional recommendations. Adv Nutr 5, 430–446. 25022992 10.3945/an.114.006122 PMC4085191

[11] Monteiro CA , Cannon G , Moubarac JC et al. (2018) The UN decade of nutrition, the NOVA food classification and the trouble with ultra-processing. Public Health Nutr 21, 5–17. 28322183 10.1017/S1368980017000234 PMC10261019

[12] Wingrove K , Lawrence MA & McNaughton SA (2021) Dietary patterns, foods and nutrients: a descriptive analysis of the systematic reviews conducted to inform the Australian dietary guidelines. Nutr Res Rev 34, 117–124. 32779564 10.1017/S0954422420000190

[13] Jukola S (2018) On the evidentiary standards for nutrition advice. Stud Hist Philos Biol Biomed Sci 73, 1–9. 29866402 10.1016/j.shpsc.2018.05.007

[14] Scrinis G (2013) Nutritionism: The Science and Politics of Dietary Advice. New York, NY: Columbia University Press.

[15] Britten P , Haven J & Davis C (2006) Consumer research for development of educational messages for the Mypyramid food guidance system. J Nutr Educ Behav 38, S108–S123. 17116589 10.1016/j.jneb.2006.08.006

[16] Álvarez Sánchez C (2019) A global review of food-based dietary guidelines. Adv Nutr 10, 590–605. 31041447 10.1093/advances/nmy130 PMC6628851

[17] Quinn M , Jordan H & Lacy-Nichols J (2021) Upstream and downstream explanations of the harms of ultra-processed foods in national dietary guidelines. Public Health Nutr 24, 5426–5435. 34392856 10.1017/S1368980021003505 PMC10195428

[18] Lee A , Fjeldsoe B , Cullerton K et al. (2019) A Rapid Review of Evidence: Discretionary Food and Drinks (Phase Two): Definition of ‘Unhealthy’ Choices and Review of Food Classification Systems. St Lucia, QLD, Australia: The University of Queensland.

[19] WorldCat (2021) WorldCat. https://www.worldcat.org/ (accessed October 2021).

[20] Hadjikakou M & Baker P (2020) The untenable role of “junk food” in a healthy and sustainable food system. In Healthy and Sustainable Food Systems, pp. 160 [ M Lawrence and S Friel , editors]. Oxon: Routledge.

[21] Lawrence M (2017) Rethinking the translation of nutrition evidence into public health practice. J Nutr Intermed Metab 8, 62.

[22] Smith K (2013) The politics of ideas: the complex interplay of health inequalities research and policy. Sci Public Policy 41, 561–574.

[23] Deutsche Gesellschaft fuer Ernaerhrung (2022) History of the German Nutrition Society. https://www.dge.de/wir-ueber-uns/geschichte/?L=0 (accessed January 2021).

[24] Hadjikakou M & Wiedmann T (2017) Chapter 12: Shortcomings of a growth-driven food system. In Handbook on Growth and Sustainability, pp. 256–276 [ PA Victor and B Dolter , editors]. Cheltenham: Edward Elgar Publishing.

[25] Keys AKM (1959) Eat Well & Stay Well. Garden City, NY: Doubleday.

[26] Keys A , Aravanis C , Blackburn HW et al. (1966) Epidemiological studies related to coronary heart disease: characteristics of men aged 40–59 in seven countries. Acta Med Scand Suppl 460, 1–392. 5226858

[27] Select Committee on Nutrition and Human Needs (1977) Dietary Goals for the United States. Washington, DC: United States Department of Agriculture.

[28] FAO & WHO (1992) World Declaration and Plan of Action for Nutrition. Italy: FAO & WHO.

[29] Pekcan G (2006) Food and nutrition policies: what’s being done in Turkey. Public Health Nutr 9, 158–162. 16512964 10.1079/phn2005939

[30] FAO & WHO (1998) Preparation and Use of Food-Based Dietary Guidelines/Report of a Joint FAO/WHO Consultation. Geneva: World Health Organization. 9795598

[31] Kennedy E & Davis CA (2000) Dietary guidelines 2000 – the opportunity and challenges for reaching the consumer. J Am Diet Assoc 100, 1462–1465. 11138437 10.1016/S0002-8223(00)00409-0

[32] Burlingame B (2004) Holistic and reductionist nutrition. J Food Compos Anal 5, 585–586.

[33] Hadjikakou M (2017) Trimming the excess: environmental impacts of discretionary food consumption in Australia. Ecol Econ 131, 119–128.

[34] Ministry of Health Brazil (2015) Dietary Guidelines for the Brazilian Population. Brasilia, Brazil: Ministry of Health Brazil.

[35] FAO & WHO (2019) Sustainable Healthy Diets. https://www.fao.org/3/ca6640en/ca6640en.pdf (accessed March 2021).

[36] The Scientific Group for the UN Food Systems Summit (2021) Science and Innovations for Food Systems Transformation and Summit Actions. Rome: United Nations.

[37] Gibney MJ , Forde CG , Mullally D et al. (2017) Ultra-processed foods in human health: a critical appraisal. Am J Clin Nutr 106, 717–724. 28793996 10.3945/ajcn.117.160440

[38] Juul F , Vaidean G & Parekh N (2021) Ultra-processed foods and cardiovascular diseases: potential mechanisms of action. Adv Nutr 12, 1673–1680. 33942057 10.1093/advances/nmab049 PMC8483964

[39] Hall KD , Ayuketah A , Brychta R et al. (2019) Ultra-processed diets cause excess calorie intake and weight gain: an inpatient randomized controlled trial of ad libitum food intake. Cell Metab 30, 67.e63–77.e63. 31105044 10.1016/j.cmet.2019.05.008 PMC7946062

[40] Elizabeth L , Machado P , Zinöcker M et al. (2020) Ultra-processed foods and health outcomes: a narrative review. Nutrients 12, 1955. 32630022 10.3390/nu12071955 PMC7399967

[41] Rockström J , Edenhofer O , Gaertner J et al. (2020) Planet-proofing the global food system. Nat Food 1, 3–5.

[42] Chea M & Mobley AR (2020) Interpretation and understanding of the dietary guidelines for Americans consumer messages among low-income adults. J Am Coll Nutr 39, 63–71. 31084517 10.1080/07315724.2019.1610918

[43] King L , Watson WL , Chapman K et al. (2012) Do we provide meaningful guidance for healthful eating? An investigation into consumers’ interpretation of frequency consumption terms. J Nutr Educ Behav 44, 459–463. 22591579 10.1016/j.jneb.2011.12.004

[44] Ares G , Vidal L , Allegue G et al. (2016) Consumers’ conceptualization of ultra-processed foods. Appetite 105, 611–617. 27349706 10.1016/j.appet.2016.06.028

[45] Aguirre A , Borneo MT , El Khori S et al. (2019) Exploring the understanding of the term “ultra-processed foods” by young consumers. Food Res Int 115, 535–540. 30599975 10.1016/j.foodres.2018.09.059

[46] Institute of Public Health (2014) Guidelines for Nutrition of the Population in the Republic of Macedonia (Translated). Skopje, North Macedonia: Institute of Public Health.

[47] Debras C , Chazelas E , Srour B et al. (2022) Artificial sweeteners and cancer risk: results from the NutriNet-Santé population-based cohort study. PLoS Med 19, e1003950. 35324894 10.1371/journal.pmed.1003950 PMC8946744

[48] NHMRC (2003) Dietary Guidelines for Australian Adults. Canberra: Commonwealth of Australia.

[49] Gezond Leven (2022) Food Outside the Food Triangle. https://www.gezondleven.be/themas/voeding/voedingsdriehoek/overige-producten (accessed January 2022).

[50] Ministry of Health & National Centre of Public Health Protection (2006) Food Based Dietary Guidelines for Adults in Bulgaria. Sofia: Ministry of Health.

