# Supplementary material for: From harmful nutrients to ultra-processed foods: exploring shifts in ‘foods to limit’ terminology used in national food-based dietary guidelines
Source: Public Health Nutr. 2022 Dec 2;26(11):2539–50. doi: 10.1017/S1368980022002580 (PMC10641640; doi:10.1017/S1368980022002580)
Supplement: Supplementary file 1 [file S1368980022002580sup.zip › S1368980022002580sup002.docx]

**Supplementary Figure 1**: Impact of agency offering funding or intellectual support on terms used to describe ‘foods to limit’ in dietary guidelines

*See the excel file for supplementary Tables 1 & 2.*
